# Chromosome 14 MicroRNA Cluster Members in Brain-Derived Extracellular Vesicles Can Be Genotype-Associated Biomarker Candidates for Alzheimer’s Disease and Frontotemporal Dementia

**DOI:** 10.3390/biom16101468

**Published:** 2026-10-09

**Authors:** Joseph Morgan, Toby Aarons, Gemma Lace, Arijit Mukhopadhyay

**Affiliations:** 1 Translational Medicine Laboratory, Biomedical Research and Innovation Centre, University of Salford, Manchester M5 4WT, UK; 2 Faculty of Science Engineering and Environment, University of Salford, Room G07b Peel Building, The Crescent, Manchester M5 4WT, UK; 3 Faculty of Health Community and Life Sciences, University of Salford, Room 322 Peel Building, The Crescent, Manchester M5 4WT, UK

**Keywords:** Alzheimer’s, frontotemporal dementia, brain-derived, extracellular vesicles, C14MC, biomarkers, microRNA

## Abstract

Background: The chromosome 14 microRNA cluster (C14MC) is the second largest microRNA (miRNA) cluster in the human genome, which is tightly regulated during early development and dysregulated in many disease conditions. Members of this cluster are reported to have important temporal and spatial roles in early development, especially in neurogenesis. Although extensively studied as cancer biomarkers, their potential as molecular signatures of neurodegenerative diseases (NDs) remains poorly understood. Given the importance of non-invasive or less-invasive biomarkers of dementia, brain-derived small extracellular vesicles (BDsEVs) represent a promising source of disease-associated miRNAs. BDsEVs can traverse the blood–brain barrier and potentially become a surrogate for molecular changes within the brain in accessible body fluids. We investigated whether BDsEV-associated C14MC miRNAs exhibit shared or disease-specific expression patterns in Alzheimer’s disease (AD) and genetically defined subtypes of frontotemporal dementia (FTD). Methods: BDsEVs were isolated from post-mortem frontal cortex tissue of neurologically healthy controls, AD, and FTD cases carrying pathogenic *GRN*, *MAPT*, or *C9orf72* mutations. Seven candidate C14MC miRNAs were quantified by RT-qPCR, while small RNA-sequencing data were analysed to characterise C14MC expression patterns. Results: Among the RT-qPCR candidates, miR-323a-5p and miR-758-5p showed significant differences between diagnostic groups. MiR-323a-5p demonstrated disease-associated upregulation in FTD-*MAPT* compared with all other groups (fold change = 6.68, adjusted *p* < 0.001) in the relative-expression analysis, with ΔCt analysis supporting all comparisons except AD. Exploratory small RNA-sequencing identified nominal expression differences in FTD-*MAPT* (miR-1197, miR-381-3p, miR-299-3p) and FTD-*C9orf72* (miR-485-3p, miR-381-3p) compared to controls, alongside nominal differences observed in AD against FTD mutation groups (miR-1247-5p), with recurrent alterations across multiple comparisons. Further nominal differences were observed between underlying proteinopathies. Conclusions: Collectively, these findings identify disease- and genotype-associated patterns of C14MC miRNAs within BDsEVs, although the sequencing findings remain exploratory and require validation in larger independent cohorts. Thus, our results highlight the potential of individual C14MC members as BDsEV-derived molecular signatures and candidate biomarkers for molecular stratification of dementia.

## 1. Introduction

Non-coding RNAs (ncRNAs) are regulatory molecules that act through epigenetic mechanisms to fine-tune gene expression at the transcriptional and post-transcriptional levels, collectively altering complex biological processes and systems [1]. One major class of well-studied ncRNAs are microRNAs (miRNAs), small transcripts of around 18–25 nucleotides in length that post-transcriptionally regulate gene expression, usually through the targeting of mRNAs, leading to a downregulation in their expression. Alongside their functional role as post-transcriptional regulators, microRNAs are reported to have major diagnostic potential as circulating biomarkers of various diseases, such as cancers, cardiovascular diseases and autoimmune conditions [2]. Amongst these, clustered miRNAs have emerged as particularly interesting diagnostic candidates, because coordinated changes across multiple miRNAs may better reflect disease-associated regulatory perturbations than individual transcripts [3].

Though studies show that individual miRNAs can alter a disease course, due to the heterogeneous and multifactorial complexities of diseases, measuring panels of miRNAs may better capture coordinated regulatory alterations, providing stronger indications of pathogenicity [4,5]. Additionally, many miRNAs act together to regulate common biological pathways, meaning that small but consistent alterations across multiple miRNAs may better reflect disease-related biological changes than alterations in individual miRNAs. Located within the imprinted human 14q32.31 locus of the DLK1-DIO3 domain, the chromosome 14 miRNA cluster (C14MC) is unique to placental mammals and the second largest miRNA cluster within the human genome, consisting of around 50 different miRNAs [6]. The cluster shows enriched expression in the brain and functions in various neurodevelopmental processes through the regulation of dendritic morphology, influencing neural plasticity and synaptic formation [7,8,9].

As the C14MC miRNAs have important neuronal functions, their role as a tumour-suppressive miRNA cluster is also well-documented, where their expression is strongly linked to limiting cancer cell proliferation and differentiation [10]. Interestingly, a pattern of downregulation of C14MC miRNAs within brain cancers such as glioblastomas, oligodendrogliomas and medulloblastomas has been observed [11,12,13], where the overexpression of C14MC in cell models restored pathways and reduced cancer cell migration and invasion. However, while the role and regulatory patterns of C14MC are well investigated within cancers, their relationship within neurodegenerative diseases (NDs) is not as well documented.

Alzheimer’s disease (AD), the most prevalent neurodegenerative disease globally and the most common cause of dementia, is characterised by amyloid beta (Aβ) aggregate formations and consequential tau tangles, both of which lead to neurodegeneration and dementia-linked symptoms such as cognitive decline, behavioural changes and memory impairment [14]. Frontotemporal dementia (FTD), the second most common cause of early-onset dementia, is defined by the degeneration of the frontal and temporal lobes, causing partially overlapping dementia-linked symptoms including confusion, speech impairment and motor dysfunction [15]. Whilst AD and FTD are separate diseases, they share many pathological similarities, adding layers of complexity in disease differentiation at early stages. For example, though AD can be characterised by Aβ assemblies and tau tangles, FTD encompasses multiple underlying proteinopathies, most commonly involving TDP-43 inclusions; however, the presence of pathological tau formations occurs in both AD and FTD [16].

As of 2019, it has been reported that around 57 million individuals had dementia globally, and by 2050, it is projected that this figure will increase to 153 million individuals [17]. Furthermore, while recent developments in therapeutics to treat AD have become available, improved methods of diagnosis for both AD and FTD are still warranted to diagnose and stratify early enough to benefit from such treatments. Currently, a focus on the source of diagnostic biomarkers has shifted to small extracellular vesicles (sEVs), membrane-bound particles that are released by cells to enable cell–cell communication [18]. These are of particular interest as, due to their ability to cross the blood–brain barrier, they can be found within biofluids including blood plasma, cerebrospinal fluid, saliva and urine, meaning they can be retrieved less invasively than through other sampling techniques. We have recently described an improved process of isolating, characterising and quantifying the microRNA cargo of these sEVs from human brain [19] and showed that such brain-derived small extracellular vesicle (BDsEV)-associated microRNAs linked to autophagy can potentially distinguish different forms of dementia [20].

It is thought that certain NDs and cancer are potential biological mirror images of one another. Firstly, NDs are characterised by continual neuronal cell death, whereas cancer cells exhibit excessive cell survival [21,22]. Secondly, in cancer, defects in DNA damage sensing and repair mechanisms permit the accumulation of genomic alterations that support cell proliferation; in comparison, neurons in NDs often exhibit the heightened activation of DNA damage response pathways, contributing to neuronal cell death [23,24]. Thirdly, in NDs, proteostatic imbalances are caused through the impairment of autophagic mechanisms, leading to the further build-up of neurotoxic aggregates, whereas in cancers, proteostatic imbalances are caused through exaggerated protein turnover and improved oxidative stress tolerance [25,26].

Given the coordinated regulation of C14MC reported in neurodevelopment and tumour suppression, together with the opposing biology of brain cancers and neurodegeneration, we hypothesised that NDs may exhibit distinct patterns of C14MC dysregulation. To test this, we investigated both cluster-level behaviour and individual C14MC miRNA cargoes within BDsEVs, isolated from fresh-frozen post-mortem brain tissue of individuals with AD and FTD with the three different mutational backgrounds in *GRN*, *MAPT* and *C9orf72*.

## 2. Materials and Methodology

### 2.1. Post-Mortem Human Brain Tissue and Ethical Considerations

Fresh-frozen post-mortem human frontal lobe samples from five diagnostic groups: 5 control, 5 AD, and three genetically defined FTD groups comprising 5 *GRN*-associated FTD (FTD-*GRN*), 5 *MAPT*-associated FTD (FTD-*MAPT*) and 5 *C9orf72*-associated FTD (FTD-*C9orf72*); were provided by the Manchester Brain Bank [27], which were collected, dissected and stored using protocols established by the BrainNet Europe consortium [28]. Each AD and FTD sample was assessed for clinical and pathological diagnosis [29,30,31], including APOE genotyping and Braak stage evaluation (Table 1). Additionally, control samples had no clinical diagnosis, with pathological assessment showing age-related changes only. Both male and female donors were included to ensure representation of both sexes across the diagnostic groups, particularly given the recognised influence of biological sex on AD prevalence in females and disease-related processes [32]. However, owing to the limited sample size, the present study was not designed or powered to investigate sex-specific differences in BDsEV miRNA expression. Further donor-level information (including age, sex, post-mortem delay, mutation/APOE status/Braak Stage, neuropathology) can be found in Supplementary Data S1.

Ethical approval was obtained from the Manchester brain bank for the storage and research use of fresh-frozen human brain tissue samples (REC reference 09/H0906/52), with local ethical approval for this project granted by the University of Salford ethics board (reference 0754).

Whilst five post-mortem brain tissue samples were available per diagnostic group, subsets of three biological samples per diagnostic group were used for individual RT-qPCR and small RNA-sequencing analyses, with samples not necessarily identical across assays. The donor cohort, EV preparations and small RNA-sequencing dataset used in the present study were previously reported in Morgan et al. (2026) [20]. The present study represents a new C14MC-focused analysis of these sequencing data. The characterisation of BDsEVs and C14MC RT-qPCR experiments presented here are newly reported and have not previously been published.

### 2.2. Isolation of Brain-Derived Small Extracellular Vesicles

For the isolation of BDsEVs from fresh-frozen post-mortem human brain tissue, the protocol described in Morgan et al. (2024) [19] was used. To begin, 250 mg frozen brain tissue was sliced and placed in 2 mL 75 U/mL collagenase type-III/Hibernate-E solution (StemCell Technologies, Cambridge, UK, Cat No. 07422; Gibco, Cat No. A1247601). Samples were incubated at 37 °C for 20 min (at the 5 min point, samples were inverted twice; at the 10 min point, samples were gently pipetted up and down twice with a 10 mL stripette), whereafter samples were added back to ice, and 20 µL 100X Halt^TM^ protease inhibitors (ThermoFisher, Loughborough, UK, Cat No. 78438) and PhosSTOP inhibitors (Roche, Burgess Hill, UK, Cat No. 4906845001) were added. After a centrifugation step (300× *g*, 10 min, 4 °C), the supernatants were collected and centrifuged further (2000× *g*, 15 min, 4 °C), where again the supernatants were slowly filtered using a 0.22 µm syringe filter (Merck, Gillingham, UK, Cat No. SLGSR33SS). Prior to size exclusion chromatography (SEC), the samples underwent a primary centrifugation step (10,000× *g*, 48 min, 4 °C), whereafter Exo-Spin^TM^ buffer (Cell Guidance Systems, Cambridge, UK, Cat No. EX06) was added to each sample (2:1) and incubated overnight at 4 °C. Each mixture was centrifuged (10,000× *g*, 96 min, 4 °C), and the pellets were then resuspended in 100 µL EV-free PBS, which was subsequently added to separate ExoSpin^TM^ columns (Cell Guidance Systems, Cambridge, UK, Cat No. EX03) and allowed to enter the column through gravity. To each column, 180 µL EV-free PBS was added, and each column was then centrifuged at 50× *g* for 1 min, eluting the BDsEVs.

### 2.3. Characterisation of Brain-Derived Small Extracellular Vesicles

Three methods were used to characterise the isolated EVs in accordance with the minimal information for studies of extracellular vesicles (MISEV) guidelines [33,34].

To identify characteristic markers on the surface and within the BDsEVs (alongside the absence of cell-derived negative markers), Western blotting was used. On 10% SDS-PAGE gels, 20 µg EV sample (in 4× loading buffer, heated at 70 °C) was loaded and run (in 1× Tris-Glycine-SDS) at 100 V before being transferred to nitrocellulose membranes (in 1× Tris-Glycine-MeOH). To confirm the true absence of the negative control calnexin within EVs, whole brain homogenates (BH) were run alongside EV samples probed for Calnexin.

Membranes were blocked in 5% BSA or 5% milk, where applicable, for 1 h, incubated with primary antibodies overnight (4 °C), and then incubated with secondary antibodies for 2 h at room temperature (antibody details and dilutions provided in Supplementary Table S1). Each membrane was developed with SuperSignal^TM^ West Atto Ultimate Sensitivity Chemiluminescent Substrate solution (Fischer Scientific, Leicestershire, UK, Cat No. 17181819) and imaged on a LI-COR Odyssey Fc Imager.

For nanoparticle tracking analysis (NTA), particle size was measured using light-scattering mode on the ZetaView Nanoparticle Analyser (Analytik, Cambridge, UK, ZetaView Software v8.06.01), where samples were first diluted 1:1000 in PBS and read at 520 nm.

Transmission electron microscopy (TEM) was used to provide another dimension of size and morphology characterisation of sEVs, where samples were sent to the University of Liverpool to be processed and imaged using TEM. For this, a 10 µL sample was fixed in 1% glutaraldehyde, washed with ddH_2_O, stained with 1% uranyl acetate and incubated in 4% uranyl acetate/2% methyl cellulose (1:9) at 4 °C for 10 min. After, samples were dehydrated using graded alcohols and imaged using the FEI 120 kV Tecnai G2 Spirit BioTwin TEM (FEI, Hillsboro, OR, USA).

### 2.4. Isolation of RNA Samples from Brain-Derived Small Extracellular Vesicles

In preparation for RNA isolation, BDsEV samples were treated with proteinase K and RNase A to reduce co-isolated EV protein and RNA prior to downstream RNA isolation. To 50 µL BDsEV sample, 1 µL 20 µg/µl proteinase K (ThermoFisher, Loughborough, UK, Cat No. 25530049) was added and incubated at 37 °C for 30 min, with subsequent incubation on ice and the addition of 0.5 µL 100× Halt^TM^ and PhosSTOP^TM^ to stop any reaction. After, to each sample, 1 µL 10 µg/µL RNase A (ThermoFisher, Loughborough, UK, Cat No. EN0531) was added and incubated at 37 °C for 30 min. Total RNA isolation was performed using an RNeasy RNA isolation kit (Qiagen, Manchester, UK, Cat No. 74004), where miRNA concentrations were quantified using a Qubit^TM^ microRNA Assay Kit (ThermoFisher, Cat No. Q32880) and Qubit^TM^ 3 Fluorometer (ThermoFisher, Loughborough, UK, Cat No. Q33230) (miRNA assay setting) and compared with Proteinase K/RNase A untreated EV samples to assess the retention of RNA protected from extracellular enzymatic degradation [19].

### 2.5. Reverse-Transcriptase Quantitative PCR

To analyse specific C14MC candidates, reverse-transcriptase quantitative PCR (RT-qPCR) was performed. The QuantimiR RT small RNA quantification kit (System Biosciences, Cambridge, UK, Cat No. RA420A-1) was used for RNA-to-cDNA conversion. To a thin-walled PCR tube, 5 µL total RNA (10 ng per sample), 2 µL 5× PolyA Buffer, 1.5 µL 5 mM ATP, 1 µL 25 mM MnCl_2_, 1 µL spike-in cel-miR-39 solution (Qiagen, Manchester, UK, Cat No. 339390, 8 × 10^9^ copies/ml) and 0.5 µL PolyA polymerase were added and incubated at 37 °C for 30 min using a thermocycler (T100 Thermal Cycler, Bio-Rad Laboratories, Watford, UK). Thus, equal total RNA input (10 ng) was used across biological samples, with an equivalent amount of exogenous cel-miR-39 added to each reaction as a technical reference for subsequent RT-qPCR normalisation. As cel-miR-39 was added following RNA extraction, it served as a technical reference for subsequent reverse-transcription and qPCR procedures rather than for EV isolation or RNA recovery. Furthermore, as cel-miR-39 is exogenous to human samples, its use provided a consistent technical reference without assuming stable expression of an endogenous miRNA across diagnostic groups, for which no universally accepted EV-associated miRNA reference has been established.

After incubation, to each sample, 0.5 µL oligo-dT adapter was added and incubated at 60 °C for 5 min, and then cooled at room temperature for 2 min. A MasterMix of 4 µL 5× RT buffer, 2 µL dNTP mix, 1.5 µL 0.1 M DTT, 1 µL reverse transcriptase, and 0.5 µL RNase-free H_2_O was added to each sample, and the mixtures were heated at 42 °C for 1 h and subsequently heated at 95 °C for 10 min, generating cDNA samples. For RT-qPCR, the generated cDNA samples were diluted 1:10 in RNase-free water.

Per RT-qPCR reaction, 15 µL 2× SYBR Green RT-qPCR MasterMix buffer (Meridian Bioscience, Cincinnati, OH, USA), 1 µL 10 µM universal reverse primer (System Bioscience, Cambridge, UK, Cat No. RA420AU-3), 1 µL 10 µM miRNA target-specific forward primer (Eurofins, Wolverhampton, UK; Supplementary Table S2), 12.5 µL RNase-free water and 1 µL 10 ng cDNA were added and mixed. RT-qPCR was performed using 40 cycles, each consisting of a denaturation step at 95 °C for 30 s, followed by a combined annealing and extension step at 60 °C for 60 s. No-template controls (NTCs) were included alongside each RT-qPCR assay and showed no detectable amplification. All target reactions for biological samples produced detectable amplification across both technical replicates; therefore, no imputation or exclusion due to undetected reactions was required.

RT-qPCR reactions were performed in technical duplicates, with duplicate Ct values averaged for each biological sample (Ct_sample_) prior to analysis. For each sample, ΔCt was calculated as the mean target miRNA Ct minus the corresponding mean cel-miR-39 Ct (Ct_control_). For each miRNA, the mean ΔCt of the three control biological samples was used as the calibrator, with the ΔΔCt calculated as:

ΔCt_sample_ − mean ΔCt_control_

Relative miRNA expression was subsequently calculated using the delta–delta Ct (2^−ΔΔCt^) method [35]. Individual Ct measurements and the corresponding calculations are provided in Supplementary Data S2.

### 2.6. Small RNA-Sequencing

Small RNA-sequencing (small RNA-seq) was used to explore small RNA cargo within BDsEVs (as described in Morgan et al., 2026 [20]). The library was prepared using the small RNA-seq library preparation kit (Lexogen, Cambridge, UK, Cat No. 052.96), where both 3′ and 5′ adapters were added, alongside P5 and P7 sequences (with indices), and amplified using endpoint PCR (Supplementary Table S3). For 3′ adapter ligation, 1 µL 3′ adapter (TGGAATTCTCGGGTGCCAAGGAACTCCAGTCAC) was added to 10 ng isolated BDsEV-derived RNA, after which 12 µL ligation mix and 1 µL enzyme mix (MasterMix 1) were added and incubated at 28 °C for 1 h. After column purification, 5′ adapter ligation was completed, where the 5′ adapter was denatured (70 °C, 2 min) and used in MasterMix 2 (1 µL denatured 5′ adapter, 11 µL ligation mix and 1 µL enzyme mix) and added to each reaction. This was heated for 1 h at 50 °C, creating an RNA/cDNA library, ready for amplification. To each RNA/cDNA library, 64 µL endpoint PCR MasterMix (50 µL PCR mix, 3 µL P5 primer and 11 µL H_2_O) was added, along with 3 µL unique Sri7 index primer; each library underwent endpoint PCR and was purified using column purification. For endpoint PCR, an initial denaturation was performed at 98 °C for 30 s, followed by 22 cycles of denaturation at 98 °C for 10 s, annealing at 60 °C for 30 s and extension at 72 °C for 15 s. Amplification was concluded with a final extension at 72 °C for 10 min.

After quality and quantification analysis, using TapeStation High Sensitivity DNA 1000 (D1000) ScreenTape Assay (Agilent, Cheadle, UK, Cat No. 5067) and Qubit 1× dsDNA high sensitivity quantification kit (ThermoFisher, Loughborough, UK, Cat No. Q33230), four libraries were multiplexed, normalised according to fragment length (bp) and concentration (ng/µL). Libraries were diluted to 4 nM using PCR-grade H_2_O, denatured using 5 µL 0.2 N NaOH, and agitated and centrifuged at 280× *g* for 1 min. After an incubation for 5 min at room temperature, the denatured libraries were diluted to 20 pM using 990 µL HT1, and then further diluted to 8 pM with HT1. For each experiment, a PhiX control was used as a spike-in standard for cluster generation, sequencing and alignment. Illumina MiSeq v3 flow cells were used, with each sequencing experiment run on the Illumina MiSeq sequencer (Cambridge, UK) (50 cycles).

### 2.7. Bioinformatic Workflow

The current bioinformatic analysis was previously described in Morgan et al. (2026) [20]. For small RNA-seq, quality assessment of each run was performed using FastQC (v0.12.1) to assess base quality, GC content, sequence length distribution and adapter contamination, after which CutAdapt (v5.2) was used to remove the Illumina small RNA adapter sequence (TGGAATTCTCGGGTGCCAAGGAACTCCAGTCAC). Post-trim quality was reassessed using FastQC, with all reads passing QC retained. To map short reads (around 18–30 nt), a Bowtie2 (v2.5.4) index was built from the hg38 human reference genome (downloaded from UCSC Genome Browser). Reads were aligned to the hg38.fa reference using very sensitive mode and single-end, end-to-end alignment. SAM files were converted to sorted BAM files using samtools (v1.22.1), where alignment statistics were quantified using the flagstat function. For each sample, miRNAs were quantified using a GFF3 annotation from miRBase (https://www.mirbase.org/, accessed on 22 September 2026) with HTSeq-count (v2.0.9), after which individual sample counts were merged into a single count matrix for downstream analysis. Non-miRNA alignment summary rows were excluded prior to differential expression analysis, resulting in a matrix containing 2652 annotated human miRNAs across 15 samples. C14MC members were subsequently extracted from the complete differential expression results for C14MC-specific analysis and visualisation. The complete miRNA count matrix used for differential expression analysis is provided in Supplementary Data S3, with full DESeq2 analysis results in Supplementary Data S4.

### 2.8. Statistical Analysis

Statistical analyses were performed to analyse differences in C14MC miRNA cargoes of each diagnostic group using RT-qPCR and small RNA-seq, all being performed in R (v4.5.2) in RStudio (v2025.09.2). To assess overall C14MC expression, a composite C14MC expression score was calculated from the seven miRNAs analysed by RT-qPCR (miR-323a-5p, miR-758-5p, miR-376c-5p, miR-376a-2-5p, miR-889-5p, miR-134-5p and miR-410-5p). For each miRNA, 2^−ΔΔCt^ values were log_2_-transformed and standardised across samples as z-scores. The composite C14MC expression score for each biological sample was calculated as the arithmetic mean of the seven miRNA-specific z-scores, with each miRNA weighted equally.

$${C14MC\;Score}_{i}=\frac{1}{7}\sum_{j=1}^{7}z_{ij}$$

where *C*14*MC Score_i_* represents the composite C14MC expression score for biological sample *i*, and *z_ij_* represents the standardised log_2_ expression value of miRNA *j* in biological sample *i*.

Group differences in composite C14MC expression scores were assessed using one-way ANOVAs, and where applicable, post hoc pairwise comparisons were performed using Tukey’s Honestly Significant Difference test. Additionally, concordance in the relative expression ranking of the seven C14MC miRNAs was assessed within each diagnostic group using Kendall’s coefficient of concordance (W). For each biological sample within a diagnostic group, the seven miRNAs were ranked according to their relative expression, and W was used to quantify agreement in these rankings across biological samples, with W values approaching 1 indicating greater concordance in relative expression rankings of the seven miRNAs.

For individual RT-qPCR analysis, statistical testing was performed on the 2^−ΔΔCt^ relative-expression values for each biological donor, following averaging of technical duplicates. Group differences in BDsEV miRNA expression were assessed using one-way ANOVAs. Where applicable, post hoc pairwise comparisons were performed using two-sample t-tests based on the pooled residual variance from the one-way ANOVA, with Holm correction for multiple testing. No additional multiple-testing correction was applied across the seven preselected C14MC miRNAs, and these analyses were therefore considered exploratory. To assess the robustness of these findings to the expression scale used for statistical testing, an additional analysis was performed using ΔCt values, where again, one-way ANOVAs were performed separately for each of the seven candidate miRNAs, with the resulting omnibus *p*-values adjusted across the seven miRNAs using the Holm method. For miRNAs retaining a significant overall group effect following this correction, pairwise comparisons were performed using the pooled residual variance from the corresponding ANOVA, with Holm correction across the 10 pairwise comparisons. As log_2_-transformed 2^−ΔΔCt^ values represent a linear transformation of ΔCt, equivalent inferential results would be obtained using log_2_-transformed relative expression values.

For differential expression analysis, DESeq 2 (v1.50.2) analysis was applied to the complete miRNA count matrix. Count data were normalised using the median-of-ratios method, with dispersion estimation and model fitting performed across the complete miRNA dataset. Wald tests were used to evaluate contrasts between diagnostic groups and proteinopathies (disease vs. control; AD vs. FTD mutation; FTD mutation vs. FTD mutation; tauopathy comparisons; TDP-43-proteinopathy comparisons). For exploratory proteinopathy-based comparisons, AD and FTD-*MAPT* (*n* = 3 per group) were combined as a tauopathy group (*n* = 6), while FTD-*GRN* and FTD-*C9orf72* (*n* = 3 per group) were combined as a TDP-43-proteinopathy group (*n* = 6). These were compared with controls (*n* = 3) and directly with each other. As proteinopathy grouping was intrinsically linked to diagnosis and genetic background within this cohort, these analyses were considered exploratory and interpreted alongside the corresponding individual diagnostic group comparisons. Here, *p*-values were adjusted for multiple testing using the Benjamini–Hochberg false discovery rate (FDR) across the complete miRNA dataset for each contrast. No C14MC miRNAs remained significant at FDR < 0.05; therefore, nominally significant C14MC miRNAs (unadjusted *p* < 0.05) are reported as exploratory findings alongside their FDR-adjusted *p*-values (*p adj*). Lollipop plots were generated using ggplot2 (v4.0.3) to visualise the top 10 C14MC miRNAs with the largest positive and negative log_2_ fold changes (log_2_FC) for each comparison, with nominally significant miRNAs indicated by triangles. The R code used to reproduce the differential expression analyses and Figures is provided in Supplementary File S1.

## 3. Results

### 3.1. Brain-Derived Small Extracellular Vesicles Characterisation Analysis

**Figure 1:**
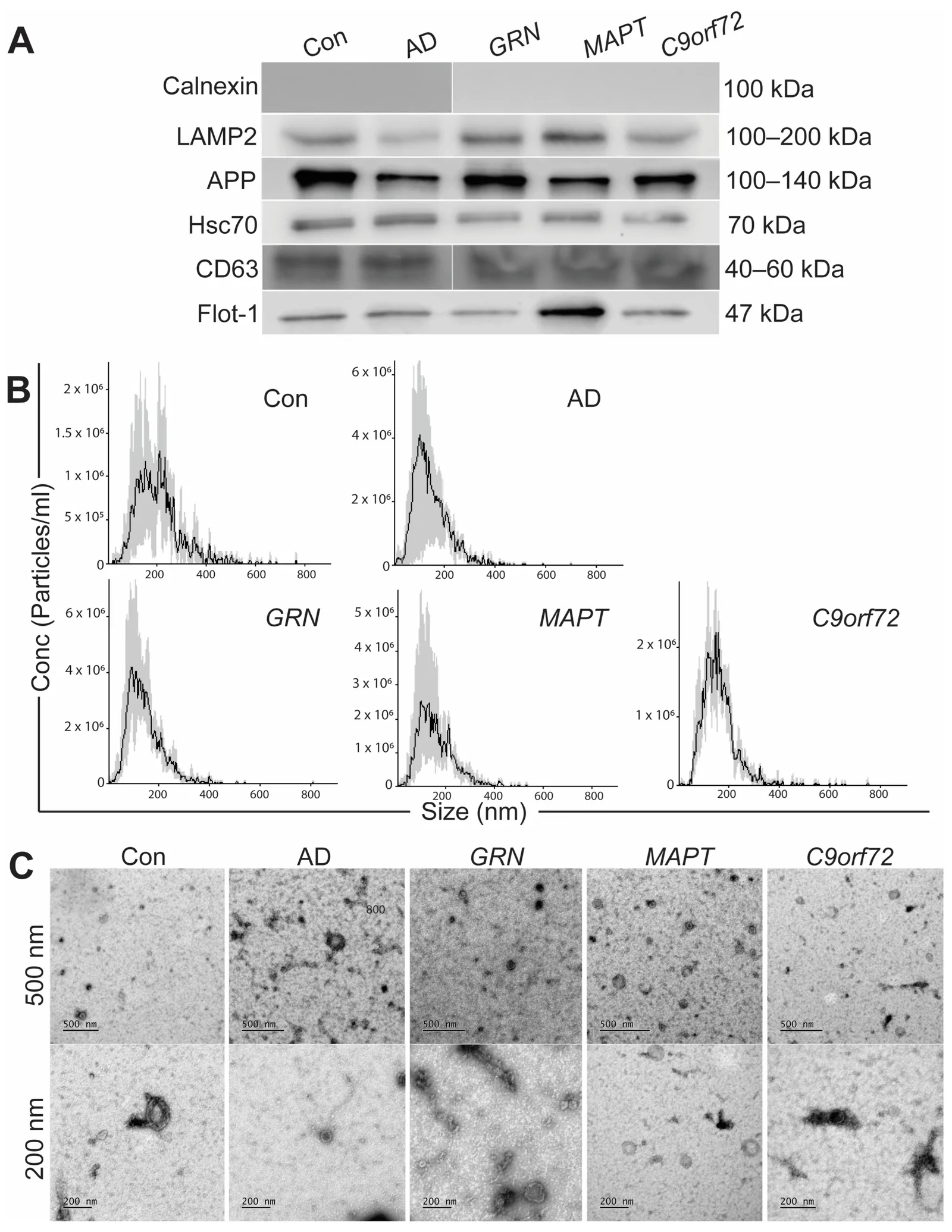
Characterisation of BDsEVs. (**A**) Western blots confirm the absence of endoplasmic reticulum-derived calnexin in BDsEVs, in contrast to the presence of sEV-specific LAMP2, Hsc70, Flot-1 and CD63, with the co-occurrence of neuron-associated APP. Original images can be found in Supplementary Figure S1. (**B**) Particle size distributions measured using NTA present the mean particle concentration (particles/mL) across biological replicates (*n* = 3, black line), with dispersion of measurements across replicates presented through shaded regions displaying the minimum and maximum values observed at each particle diameter (nm). The morphology of BDsEVs, observed using TEM (**C**), displays cup-like vesicular structures within the characteristic size of sEVs (<200 nm) in both high- and low-magnification images.

Brain-derived EVs were characterised using Western blotting (Figure 1A); five positive markers were detected, including membrane-bound CD63 and LAMP2, cytosolic Hsc70, Flot-1 and neuron-associated APP. Additionally, the absence of endoplasmic reticulum-derived calnexin was shown within BDsEV samples, but it was present within both whole BH and whole cell samples (Supplementary Figure S1). Importantly, the term BDsEVs therefore refers to EVs isolated and does not imply selective enrichment of neuron-derived EVs. Size distribution data (Figure 1B; Supplementary Figure S2) and percentage of CD-positive particles within diagnostic groups have previously been reported in Morgan et al. (2026) [20]. Most particles fell within the characteristic range of sEVs as predefined within MISEV guidelines (50–200 nm), where a few larger particle sizes could be attributed to aggregated sEVs. These findings were reinforced using TEM (Figure 1C), where not only were particles below 200 nm in diameter shown within each diagnostic group, but aggregates of sEVs were also observed. As suggested in MISEV2023 [34], high- and low-magnification images taken using TEM of particles are displayed.

### 3.2. Expression of Chromosome 14 MicroRNA Cluster Candidates Within AD and FTD Brain-Derived Small Extracellular Vesicles

To explore patterns of overall and relative C14MC miRNA expression across diagnostic groups, composite expression and concordance were examined before investigating individual miRNAs. To determine whether overall C14MC expression differed between diagnostic groups, a composite C14MC expression score was calculated; however, no significant differences were observed between diagnostic groups (*F*(4,10) = 1.22, *p* = 0.361; Supplementary Table S4). Exploratory analysis of concordance in the relative expression rankings of the seven miRNAs demonstrated low concordance in control (*W* = 0.061), AD (*W* = 0.061), FTD-*MAPT* (*W* = 0.020) and FTD-*C9orf72* (*W* = 0.245) samples (Supplementary Table S5). In contrast, FTD-*GRN* samples displayed a comparatively high concordance (*W* = 0.735; *p* = 0.0058), indicating greater similarity in the relative expression ranking of the seven miRNAs across the three biological samples. However, given the small number of biological samples analysed (*n* = 3 samples per group), these findings should be interpreted cautiously and do not establish coordinated biological regulation of the C14MC.

Given the absence of cluster-wide differences, individual RT-qPCR expression profiles were subsequently examined to determine whether specific C14MC members exhibited disease-associated dysregulation. Full RT-qPCR results, including mean fold change (FC), ±SEM, and pairwise significance, are shown in Supplementary Data S2.

**Figure 2:**
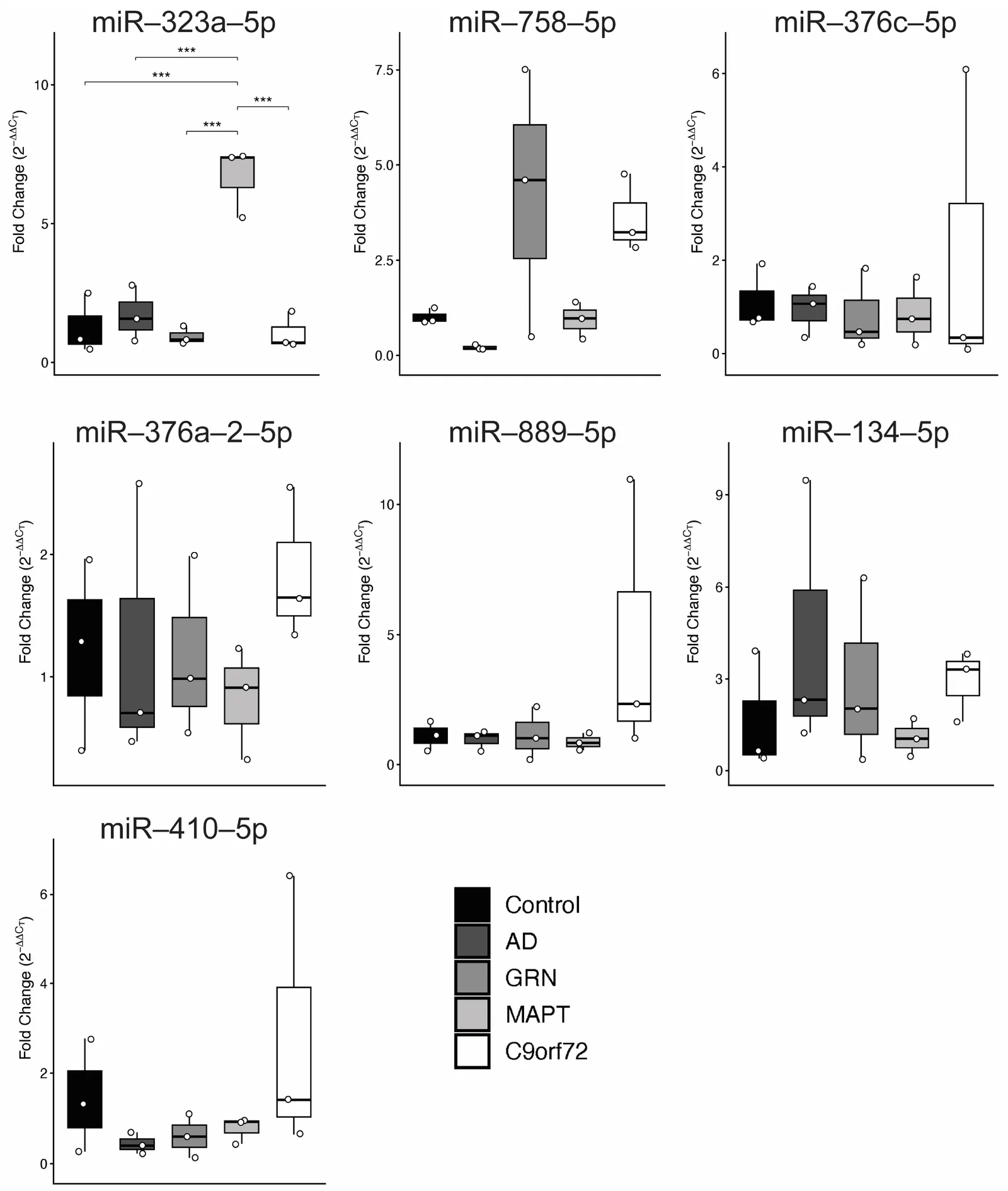
Differential expression of candidate C14MC microRNAs distinguishing different disease subtypes. Candidate C14MC members are measured across different disease conditions. Fold change (FC) was normalised against the cel-miR-39 spike-in, where each box plot shows the median and interquartile range, with whiskers extending to values within 1.5× the interquartile range and individual biological replicates displayed (*** = *p adj* < 0.001). MiR-323a-5p as BDsEV cargo was significantly upregulated (after Holm correction) in FTD-*MAPT* compared to controls (FC = 6.68, ±SEM = 0.729; *p adj* = 0.0003) as well as compared to other disease subgroups (AD *p adj* = 0.0005; FTD-*GRN p adj* = 0.0002 and FTD-*C9orf72 p adj* = 0.0002).

To explore expression differences in C14MC members between diagnostic groups, a one-way ANOVA was conducted for each miRNA, where only miR-323a-5p (*F* = 20.6675, *p* = 7.9898 × 10^−5^) and miR-758-5p (*F* = 3.4856, *p* = 0.0497) showed significant differences between the groups (Supplementary Table S6). In pairwise comparisons (after Holm corrections) within each group, only miR-323a-5p displayed significant upregulation within FTD-*MAPT* (FC = 6.68, ±SEM = 0.729), when compared to control (FC = 1.27, ±SEM = 0.624, *p adj* = 0.0003), AD (FC = 1.71, ±SEM = 0.584, *p adj* = 0.0005), FTD-*GRN* (FC = 0.944, ±SEM = 0.186, *p adj* = 0.0002) and FTD-*C9orf72* (FC = 1.07, ±SEM = 0.385, *p adj* = 0.0002) samples (Figure 2).

Additionally, miR-758-5p showed a significant overall difference between diagnostic groups. Exploratory pairwise comparisons indicated nominally increased expression in *GRN* compared with controls (FC = 4.20, ±SEM = 2.03 vs. FC = 1.01, ±SEM = 0.115, *p* = 0.0402), AD (FC = 0.207, ±SEM = 0.207, *p* = 0.0145) and *MAPT* (FC = 0.936, ±SEM = 0.280, *p* = 0.0365). Similarly, expression was nominally increased in *C9orf72* compared with AD (*p* = 0.0306). However, none of these pairwise differences remained significant following Holm correction for multiple testing.

To assess the robustness of these findings to the expression scale for statistical testing, an additional analysis was performed using ΔCt values (Supplementary Data S2). Significant overall diagnostic-group effects were observed for miR-323a-5p (*F*(4,10) = 6.61, *p* = 0.0072) and miR-758-5p (*F*(4,10) = 7.11, *p* = 0.0056), both of which remained significant following Holm correction across the seven candidate miRNAs (miR-323a-5p *p adj* = 0.0433; miR-758-5p *p adj* = 0.0391). For miR-323a-5p, Holm-adjusted pairwise comparisons remained significant between FTD-*MAPT* and control (*p adj* = 0.0175), FTD-*GRN* (*p adj* = 0.0158) and FTD-*C9orf72* (*p adj* = 0.0165), yet the FTD-*MAPT* vs. AD comparison did not remain significant (*p adj* = 0.0659). For miR-758-5p, significant pairwise differences were observed for AD vs. FTD-*GRN* (*p adj* = 0.0145) and AD vs. FTD-*C9orf72* (*p adj* = 0.0072).

### 3.3. Effect Size-Focused Analysis of C14MC miRNA Expression Across Disease Subtypes

Following differential expression analysis of the complete miRNA dataset previously reported in Morgan et al. (2026) [20], C14MC members were extracted to examine disease- and mutation-associated expression patterns within the cluster. Following Benjamini–Hochberg FDR, no C14MC miRNA reached statistical significance (*p adj* > 0.05); however, several miRNAs exhibited nominal significance (*p* < 0.05) and are therefore reported as exploratory findings. Full DESeq2 results for each comparison can be found in Supplementary Data S4.

**Figure 3:**
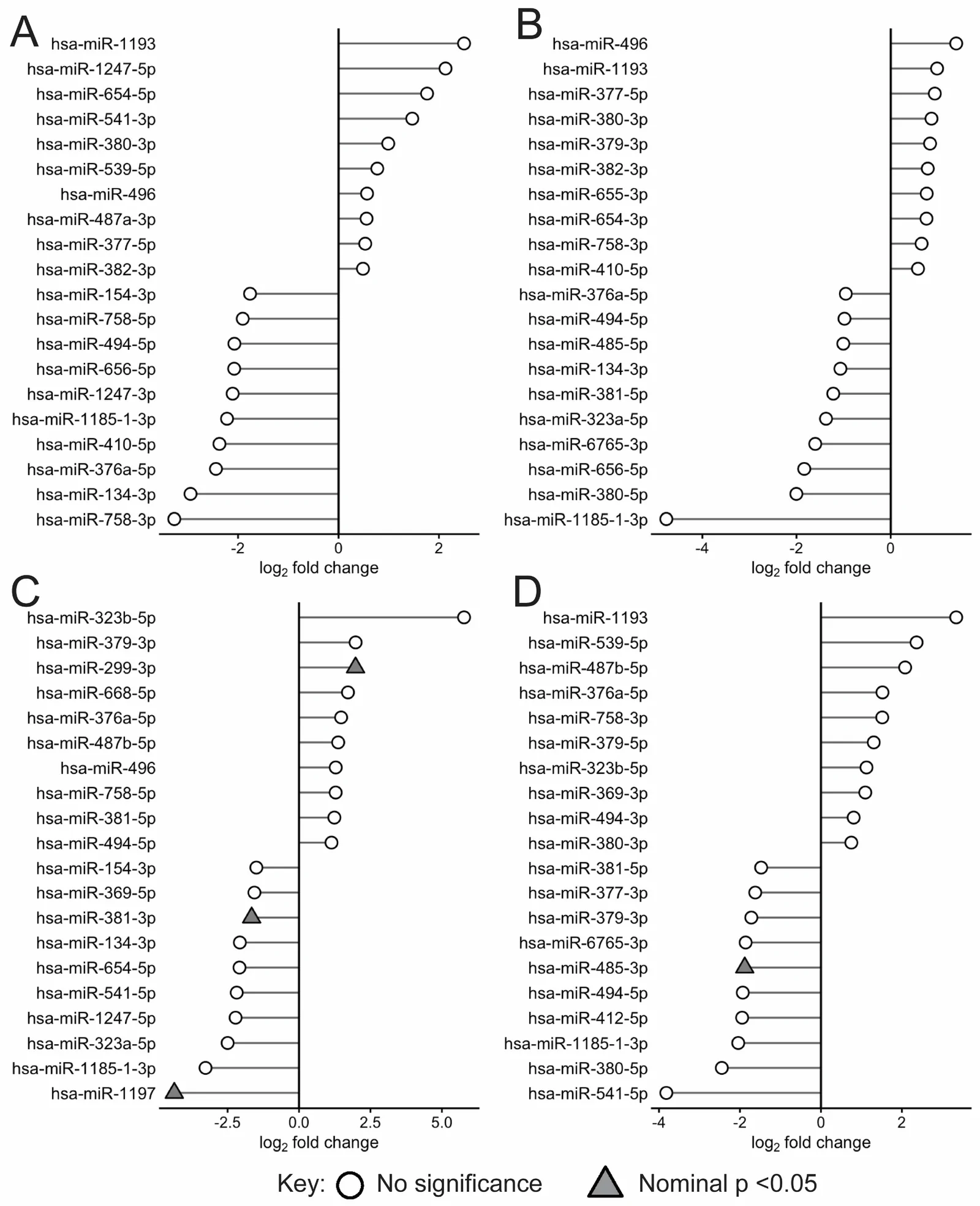
Directional expression trends of C14MC miRNAs across all disease subtypes compared to controls. Lollipop plots reveal the highest 10-ranked differentially expressed C14MC miRNAs of (**A**) AD, (**B**) FTD-*GRN*, (**C**) FTD-*MAPT* and (**D**) FTD-*C9orf72* BDsEV-associated C14MC cargoes compared to control samples, with log_2_FC plotted. DESeq2 was run in R, with Wald testing applied (biological replicates *n* = 3); non-significant (circles) and nominally significant (triangles) C14MC miRNAs are plotted (unadjusted *p* < 0.05).

For each comparison (disease vs. control; AD vs. FTD mutation; within-FTD comparisons; tauopathy vs. TDP-43-proteinopathy), lollipop plots were used to visualise log_2_ fold change (log_2_FC), where no consistent patterns were observed. Out of all diagnostic groups compared with control, only FTD-*MAPT* and FTD-*C9orf72* presented significantly nominal miRNA dysregulation. Notably, miR-381-3p was nominally decreased in both FTD-*MAPT* (log_2_FC = −1.66, *p* = 0.015, *p adj* = 0.481) and FTD-*C9orf72* (log_2_FC = −1.36, *p* = 0.045, *p adj* = 1.000) compared with controls (Figure 3), suggesting a potential shared disease-associated reduction across these two diagnostic groups. FTD-*MAPT* additionally showed a nominal decrease in miR-1197 (log_2_FC = −4.37, *p* = 0.005, *p adj* = 0.284) and a nominal increase in miR-299-3p (log_2_FC = 1.98, *p* = 0.039, *p adj* = 0.841), whereas FTD-*C9orf72* also showed a nominal decrease in miR-485-3p (log_2_FC = −1.88, *p* = 0.015, *p adj* = 1.000). However, none of these differences survived Benjamini–Hochberg FDR corrections.

**Figure 4:**
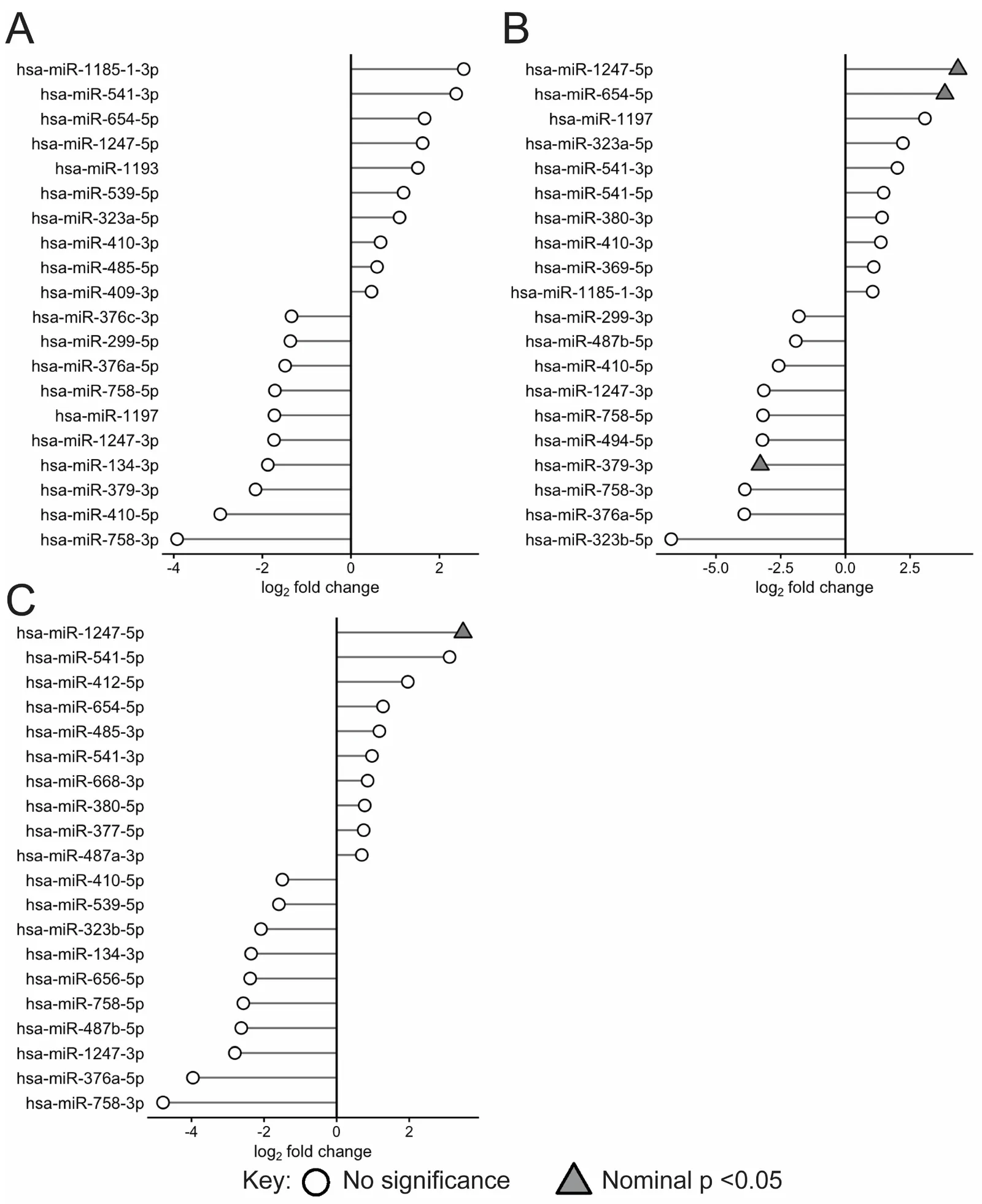
Directional expression trends of C14MC miRNAs distinguishing AD from different FTD mutational backgrounds. Lollipop plots reveal the highest 10-ranked differentially expressed C14MC miRNAs in AD samples compared to (**A**) FTD-*GRN*, (**B**) FTD-*MAPT* and (**C**) FTD-*C9orf72* BDsEV-associated C14MC cargoes, with log_2_FC plotted. DESeq2 was run in R, with Wald testing applied (biological replicates *n* = 3), where non-significant (circles) and nominally significant (triangles) C14MC miRNAs are plotted (unadjusted *p* < 0.05).

When comparing C14MC expression between AD and FTD mutations (Figure 4), several nominal differences were identified. One similarity observed when comparing AD with FTD-*MAPT* and FTD-*C9orf72* was the nominal increase in miR-1247-5p (vs FTD-*MAPT*: log_2_FC = 4.35, *p* = 0.011, *p adj* = 0.654; vs. FTD-*C9orf72*: log_2_FC = 3.48, *p* = 0.018, *p adj* = 1.000) (Figure 4B,C). Further to this, two other miRNAs showed nominal differences when comparing AD vs. FTD-*MAPT*, such as nominally increased miR-654-5p (log_2_FC = 3.85, *p* = 0.013, *p adj* = 0.658) and decreased miR-379-3p (log_2_FC = −3.30, *p* = 0.030, *p adj* = 0.990). None of these differences remained significant following FDR corrections. Moreover, no C14MC miRNAs were found to be significant in the AD vs. FTD-*GRN* comparison.

**Figure 5:**
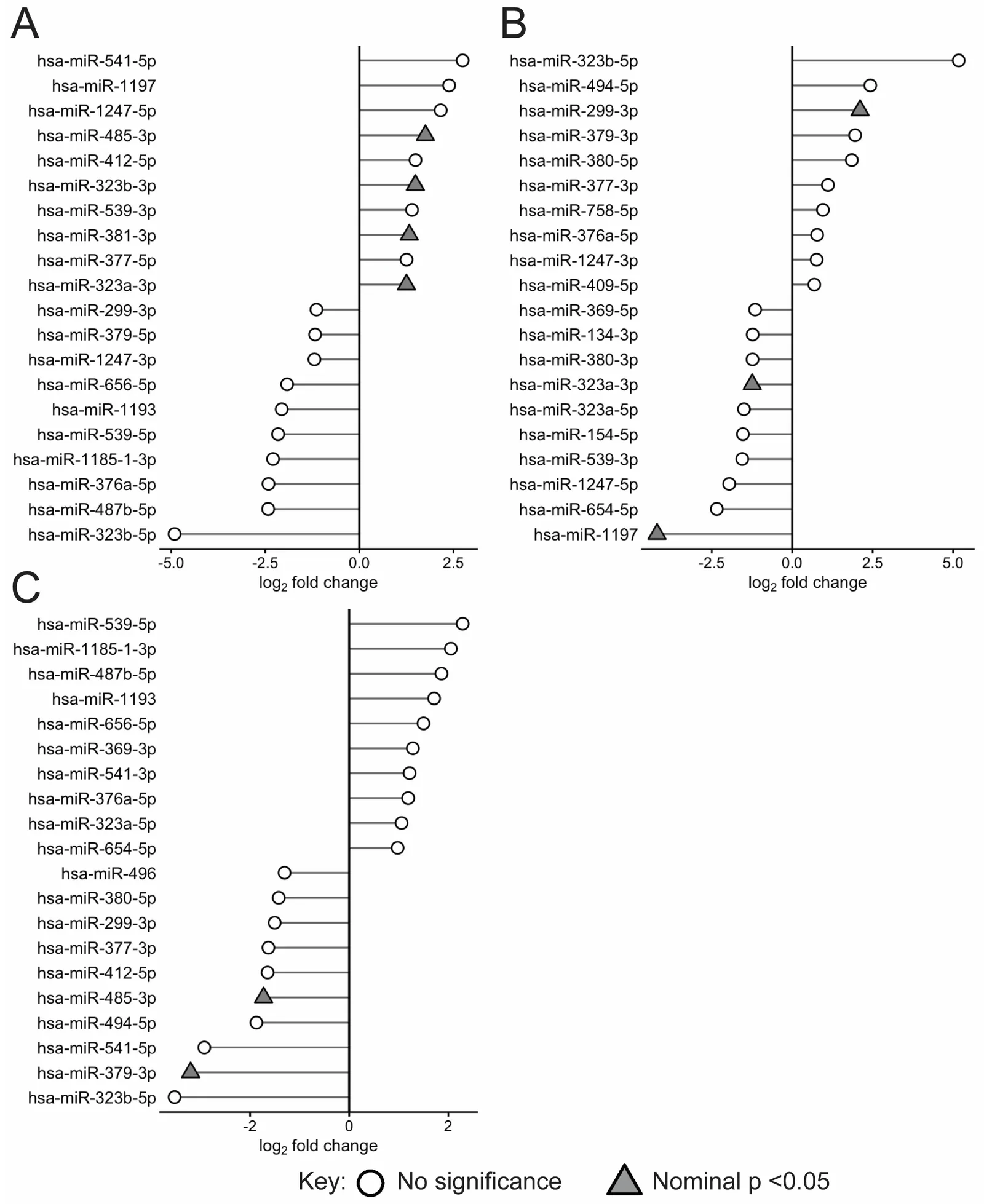
Differential C14MC miRNA expression patterns within the FTD subtypes. Lollipop plots reveal the highest 10-ranked differentially expressed C14MC miRNAs in (**A**) *GRN*, (**B**) *MAPT* and (**C**) *C9orf72* BDsEV C14MC-associated cargoes compared to the other two FTD mutation samples, with log_2_FC plotted. DESeq2 was run in R, with Wald testing applied (biological replicates *n* = 3), where non-significant (circles) and nominally significant (triangles) C14MC miRNAs are plotted (unadjusted *p* < 0.05).

Although no nominally significant C14MC differences were observed when comparing FTD-*GRN* against control, or even comparing AD vs. FTD-*GRN*, 4 nominally increased miRNAs were observed (miR-485-3p: log_2_FC = 1.76, *p* = 0.010, *p adj* = 0.975; miR-323b-3p: log_2_FC = 1.49, *p* = 0.025, *p adj* = 1.000; miR-381-3p: log_2_FC = 1.33, *p* = 0.021, *p adj* = 1.000; miR-323a-3p: log_2_FC = 1.25, *p* = 0.040, *p adj* = 1.000) when comparing FTD-*GRN* and the other two FTD genetic subgroups. Notably, miR-381-3p was also nominally decreased in both FTD-*MAPT* and FTD-*C9orf72* compared with controls. When comparing FTD-*MAPT* to the other FTD mutations (Figure 5B), miR-299-3p was nominally increased (log_2_FC = 2.11, *p* = 0.008, *p adj* = 0.792), whereas miR-1197 (log_2_FC = −4.20, *p* = 0.007, *p adj* = 0.792) and miR-323a-3p (log_2_FC = −1.24, *p* = 0.048, *p adj* = 0.994) were nominally decreased. Lastly, the two miRNAs nominally decreased in FTD-*C9orf72* when compared to the other FTD mutations were miR-379-3p (log_2_FC = −3.20, *p* = 0.010, *p adj* = 0.995) and miR-485-3p (log_2_FC = −1.73, *p* = 0.019, *p adj* = 0.995) (Figure 5C). Again, none of these differences remained significant following FDR correction.

**Figure 6:**
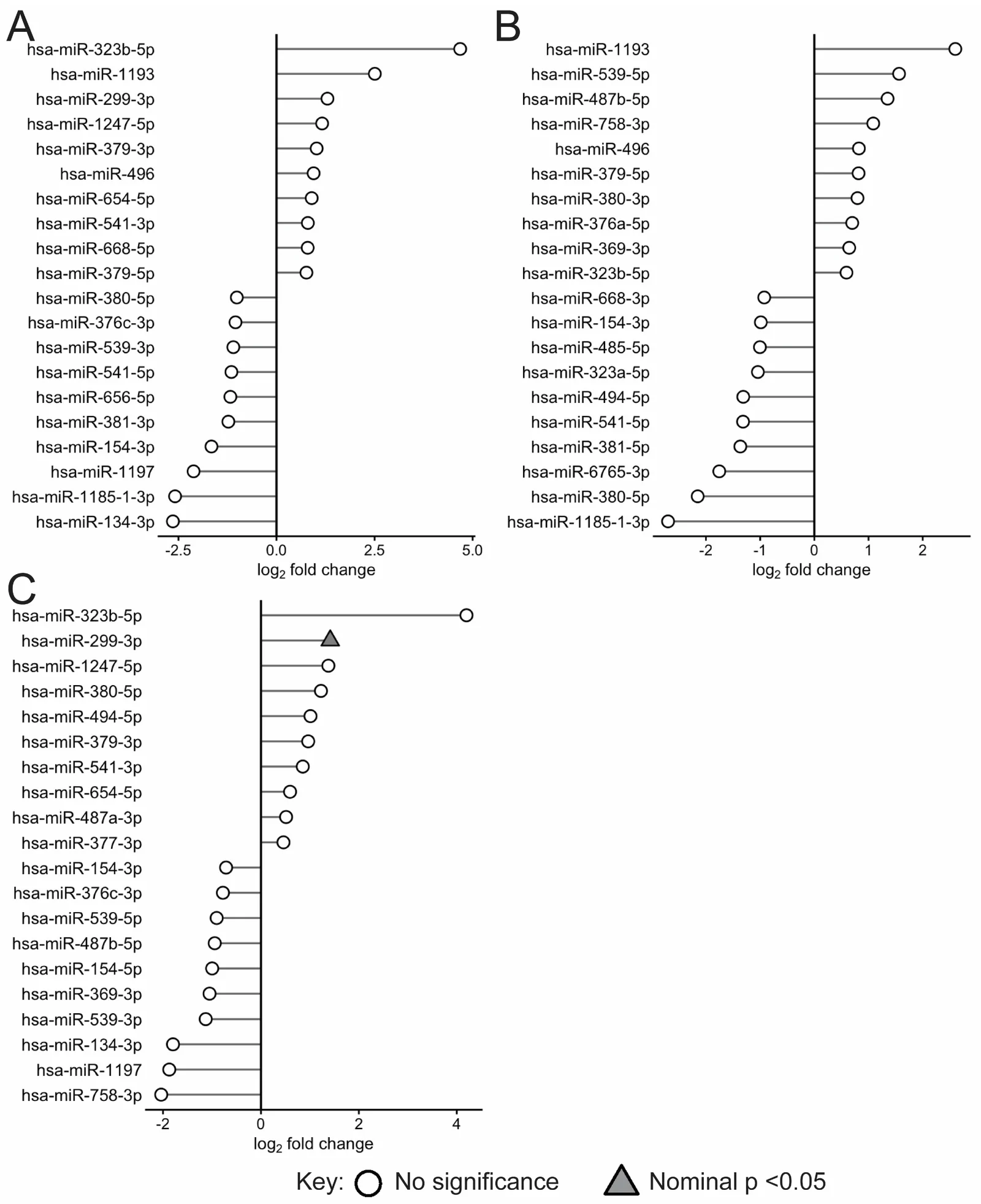
Pathology-associated C14MC miRNA expression trends across tau and TDP-43 proteinopathies. Lollipop plots reveal the highest 10-ranked differentially expressed C14MC miRNAs in (**A**) tauopathies (AD and FTD-*MAPT*, *n* = 6) vs. control (*n* = 3); (**B**) TDP-43 proteinopathies (FTD-*GRN* and FTD-*C9orf72*, *n* = 6) vs. control (*n* = 3); and when comparing (**C**) tauopathies (*n* = 6) vs. TDP-43 proteinopathies (*n* = 6), with log_2_FC plotted. DESeq2 was run in R, with Wald testing applied where non-significant (circles) and nominally significant (triangles) C14MC miRNAs are plotted (unadjusted *p* < 0.05).

To examine potential proteinopathy-associated patterns, diagnostic groups were further categorised into their respective proteinopathies (tau: AD and FTD-*MAPT*; TDP-43: FTD-*GRN* and FTD-*C9orf72*) and compared against control and each other. Whilst no nominally significant miRNAs were observed when comparing either proteinopathy group to control samples (Figure 6A,B), when comparing tauopathies against TDP-43 (Figure 6C), only miR-299-3p was nominally increased (log_2_FC = 1.40, *p* = 0.043, *p adj* = 1.000); however, this did not remain significant following FDR corrections. This observation of an increase in miR-299-3p was also observed in the individual comparisons between FTD-*MAPT* and control, and against the other FTD mutations too, suggesting that this nominal increase may predominantly be influenced by the FTD-*MAPT* subgroup.

## 4. Discussion

The C14MC represents a distinctive and evolutionarily conserved regulatory unit of placental mammals, embedded within the imprinted *DLK1-DIO3* locus. Increasing evidence indicates that C14MC is subject to coordinated epigenetic regulation and contributes to diverse biological processes, including neurodevelopment, neuronal differentiation and disease pathogenesis. Although the cluster has been extensively investigated in developmental biology and cancer, its contribution to NDs remains comparatively unexplored. In the present study, we investigated C14MC-associated miRNA expression in BDsEVs across genetically defined dementia subtypes, and despite the coordinated regulation reported for C14MC in other biological contexts, no significant differences in composite C14MC expression were detected across AD or FTD mutation groups. However, given the limited number of biological replicates, the absence of a significant difference cannot exclude broader cluster-wide alterations. Within these limitations, the observed differences among individual miRNAs suggest that specific C14MC members may contribute to disease-associated expression patterns.

Consistent with this, only a relatively small subset of validated C14MC miRNAs demonstrated disease-associated expression differences. This selective pattern is particularly notable given the genomic organisation and coordinated regulation of the cluster and suggests that individual C14MC members may have distinct sensitivities to disease-specific molecular environments. Among the validated candidates, miR-758-5p and miR-323a-5p exhibited significant overall differences between diagnostic groups. miR-758-5p is of biological interest because of its reported involvement in pathways relevant to neuronal function, neurogenesis, protein aggregation and cognitive impairment, in addition to its recognised tumour-suppressive functions. Interestingly, miR-758-5p has been reported to regulate *ZBTB20*, an important transcriptional regulator of neurogenesis, where increased miR-758-5p expression can suppress ZBTB20, with downstream effects involving GSK-3β, tau phosphorylation, and dendritic and synaptic development [36,37,38]. miR-758-5p has also been reported to target *ABCA1*, a key regulator of cellular lipid and cholesterol homeostasis with relevance to neuronal function, where reduced ABCA1 activity has been associated with cognitive deficits in animal models and with increased Aβ deposition and proteinopathy in AD [39,40,41]. These observations provide biological plausibility for a potential involvement of miR-758-5p in neurodegenerative processes. However, in the present study, the strength of the miR-758-5p associations varied according to the expression scale used for statistical analysis. While pairwise differences based on relative expression values did not remain significant following Holm correction, analysis using ΔCt values identified an overall diagnostic group effect that remained significant following Holm correction across the seven candidate miRNAs, with significant pairwise differences between AD and both FTD-*GRN* and FTD-*C9orf72*. Nonetheless, given the small number of biological samples analysed, these findings should be regarded as exploratory and require validation in substantially larger cohorts.

In contrast, miR-323a-5p demonstrated a more pronounced disease-associated pattern, with significantly increased expression in FTD-*MAPT* compared with other diagnostic groups in the 2^−ΔΔCt^ relative-expression analysis. This pattern was largely retained in the ΔCt analysis following Holm correction, with significant pairwise differences between FTD-*MAPT* and control, FTD-*GRN* and FTD-*C9orf72*, although the FTD-*MAPT* versus AD comparison did not remain significant after correction. Given the small number of biological samples analysed, these findings should be considered exploratory and require validation in larger independent cohorts.

To date, a direct association between miR-323a-5p, FTD and tau pathology has not been established. Nevertheless, previous studies implicate this miRNA in biological pathways relevant to neurodegeneration. Takamura et al. (2018) [42], for example, reported increased miR-323a-5p expression following stimulation with TNF-α, a pro-inflammatory cytokine that is consistently implicated in neuroinflammatory processes associated with neurodegenerative disease [43,44]. Furthermore, miR-323 has been implicated in the regulation of neuronal death following ischemic injury [45], a process characterised by progressive neuronal dysfunction and loss that shares several molecular pathways with neurodegenerative pathology [46]. While these findings do not establish a causal relationship between miR-323a-5p and FTD-*MAPT*, they provide a mechanistic context in which its selective enrichment in *FTD-MAPT* BDsEVs warrants further investigation, although the findings require independent validation.

Exploratory analysis of the complete C14MC repertoire in the small RNA sequencing dataset identified nominal differences in individual C14MC members across genetically and pathologically distinct forms of dementia, although none remained significant following FDR correction. In AD, nominally increased miR-1247-5p was observed in comparisons with FTD-*MAPT* and FTD-*C9orf72*, alongside nominally increased miR-654-5p and decreased miR-379-3p in comparisons with only FTD-*MAPT*. These findings are of particular interest because several of these miRNAs have previously been linked to biological processes implicated in psychiatric decline and neurodegeneration. Qiu et al. (2024) [47], for example, demonstrated an inverse relationship between EV-associated miR-379-3p and the severity of psychiatric symptoms, with a particularly strong association with anxiety, a linked symptom of NDs. Similarly, miR-1247-5p has been implicated in neuroprotection and axonal regeneration in mice [48], suggesting that its increased abundance in the present dataset could represent a biologically meaningful disease-associated alteration. Yaqub et al. (2025) [49] also reported strong associations between increased miR-654-5p and AD-associated markers, including Nfl, tau proteins and amyloid-beta monomers. These interpretations remain provisional; however, functional studies will be required to determine whether altered miRNA cargo contributes directly to disease mechanisms or instead reflects downstream responses to neurodegenerative pathology.

Nominal differences in C14MC miRNAs were also observed among the FTD subgroups, potentially reflecting differences in their underlying molecular pathologies. Recurrent disease-associated alterations across multiple comparisons were observed for miR-381-3p in FTD-*MAPT* and FTD-*C9orf72*, miR-1197 and miR-299-3p in FTD-*MAPT* and miR-485-3p in FTD-*C9orf72* across multiple comparisons. The neuroprotective role of upregulated miR-381-3p has been well-documented; in 2022, Che et al. [50] demonstrated its function in the amelioration of hypoxia/ischemic-induced neuronal damage in microglia. Additionally, miR-381-3p has also been shown to promote neural stem cell proliferation and differentiation; therefore, any decreases shown, especially within FTD mutations, may highlight potential disease-associated mechanisms for FTD pathogenesis.

Among the FTD-*MAPT*-associated miRNAs observed across multiple comparisons, miR-299-3p has previously been associated with neuronal function, displaying preferential neuronal expression within human prefrontal cortex, with predicted targets enriched in neural pathways, including axonal guidance [51]. Another function of miR-299-3p has been shown by Montalban et al. (2014) [52] as an early NGF-responsive miRNA in neuronal cells, further suggesting a potential role in neurotrophin-associated neuronal signaling; with altered abundance within FTD-*MAPT* BDsEVs, this may warrant further investigation. Furthermore, miR-1197 has been predicted to target DAAM2 and MAOA, genes that have strong links to neuronal maintenance and neurodegeneration [53,54,55]. Two other miRNAs that were identified when comparing FTD-*GRN* against the other FTD mutations were nominally increased miR-323b-3p and miR-323a-3p. Interestingly, circulating miR-323-3p has previously been shown to be associated with multimorbidity and decreased cognitive performance in middle-aged adults [56], an interesting relationship given FTD is the second most common cause of early-onset dementia. Again, further studies involving larger cohorts are needed, alongside in-depth functional examination to clarify the roles of such miRNAs within dementia-causing diseases.

Exploratory comparison according to proteinopathy groups (tauopathies vs. TDP-43-proteinopathies) showed a nominal increase in miR-299-3p, a result that was also observed in the FTD-*MAPT* vs. control comparison, suggesting this difference appeared to be predominantly influenced by the FTD-*MAPT* subgroup rather than being strongly shared across both AD and FTD-*MAPT*. As proteinopathy classification was intrinsically linked to diagnosis and genetic background within this cohort, these exploratory findings cannot be interpreted independently of the constituent diagnostic groups. Nevertheless, they support further investigation of whether individual C14MC miRNAs are associated with underlying molecular pathology in larger cohorts in which proteinopathy can be examined independently of diagnosis and genotype.

Several limitations should be considered when interpreting these findings. First, sample size represents a major constraint. Whilst five post-mortem brain tissue samples were available per diagnostic group, costs associated with downstream molecular analyses necessitated the use of subsets of three biological samples per group for individual assays. The limited number of biological replicates within individual molecular analyses reduces statistical power and increases susceptibility to inter-individual variability. Furthermore, correction for multiple testing, while essential to limit false-positive discovery, inevitably reduces sensitivity to detect relatively subtle but potentially biologically relevant changes. The present findings should therefore be considered hypothesis-generating rather than definitive. Independent replication using larger, demographically matched post-mortem cohorts will be essential to establish the reproducibility and disease-associated specificity of the exploratory C14MC expression patterns.

Second, substantial biological heterogeneity is inherent to the human brain and to neurodegenerative disease. Brain tissue is regionally specialised, with marked differences in cellular composition, molecular phenotype and susceptibility to neurodegeneration between cortical and subcortical regions and between individual cortical areas. The present study focused on frontal lobe-derived material and therefore may not capture C14MC signatures present in other brain regions. Additional sources of heterogeneity may include differences in *C9orf72* repeat expansion size, age, sex and other demographic variables, as well as disease stage and individual differences in neuropathological burden. Of particular relevance, age differed significantly between control and disease groups, representing an important potential confounder given the established effects of ageing on both neurodegeneration and extracellular vesicle biology [57]. Furthermore, although five donors were available per diagnostic group, subsets of three biological samples were used for individual molecular analyses, and the specific donor allocation to each assay could not be retrospectively reconstructed. Consequently, the extent to which differences in individual donor characteristics may have contributed to assay-specific findings cannot be determined. Given the limited number of biological replicates included within individual molecular analyses, and the substantial age differences between diagnostic groups, statistically robust adjustment for age was not considered feasible. Consequently, an influence of age on the observed miRNA expression differences, including the pronounced miR-323a-5p expression observed in FTD-*MAPT*, cannot be excluded. Consistent with the complexity of these biological influences, principal component analysis demonstrated substantial overlap between diagnostic groups [20]. Future studies should therefore incorporate larger, age- and sex-matched cohorts, multiple brain regions and sufficiently powered statistical models capable of accounting for relevant biological and technical covariates.

Third, the use of BDsEV cargo introduces additional technical considerations. EV-associated miRNAs provide an attractive means of accessing molecular information from the brain; however, BDsEV populations are heterogeneous and may originate from multiple brain-resident cell types, including neurons and glia. Consequently, changes detected in bulk BDsEV-associated miRNA profiles cannot necessarily be attributed to a specific neuronal population or interpreted as direct evidence of altered intracellular miRNA expression. Future investigations combining EV subpopulation characterisation, cell-type-specific approaches and functional validation will be important for resolving the cellular origin and biological consequences of the identified signatures. A further consideration is the discordant direction of miR-323a-5p expression between the RT-qPCR and small RNA-seq data. While RT-qPCR demonstrated increased miR-323a-5p expression in FTD-*MAPT*, the sequencing data showed an opposing, non-significant directional trend relative to controls. This discordance may reflect inter-individual or methodological variation, particularly as the samples analysed were not necessarily the same between assays. Therefore, the miR-323a-5p finding requires independent validation in a larger cohort before a consistent FTD-*MAPT*-associated expression pattern can be established. An additional technical consideration relates to RT-qPCR normalisation, and whilst equal total RNA input was used, cel-miR-39 was introduced following RNA extraction and therefore does not account for variation arising during EV isolation or RNA recovery. Furthermore, the use of equal total RNA input means that the resulting relative expression measurements may not directly represent miRNA abundance per vesicle. Normalisation to exogenous cel-miR-39 may not account for potential biological variation in EV abundance or endogenous miRNA composition between samples, consistent with the recognised challenges associated with the normalisation of EV-associated miRNA [34]. Although primer efficiencies were assessed during the initial assay optimisation, the corresponding standard curve data and melt-curve data were not retained and therefore could not be retrospectively reported. This represents an additional limitation when interpreting the miR-323a-5p RT-qPCR finding and further supports the need for independent technical and biological validation.

Notwithstanding these limitations, the present findings provide an important proof-of-concept for investigating C14MC-derived miRNAs in BDsEVs as potential molecular signatures of neurodegenerative disease. The nominal differences observed in individual miRNAs between genetically defined FTD subtypes and between different proteinopathies warrant further exploration. However, their potential clinical utility will require validation in larger independent cohorts, assessment in less-invasive biofluids such as plasma or CSF, and comparison with established clinical, genetic and neuroimaging biomarkers. Such studies will be necessary to determine the reproducibility and disease specificity of C14MC-derived molecular signatures and to establish whether they have future clinical utility as biomarkers for minimally invasive early detection, differential diagnosis or molecular stratification of neurodegenerative diseases.

Beyond their potential biomarker relevance, the present findings raise an intriguing question concerning the evolutionary and developmental significance of C14MC in neurodegeneration. Notably, miR-379-3p, one of the principal *FTD-C9orf72*-associated miRNAs identified in this study, belongs to the evolutionarily acquired miR-379/410 subcluster within C14MC, which has been implicated in the specification of cortical projection neuron identity during development [58]. The C14MC is itself a feature of placental mammalian evolution, as is the corpus callosum, the major commissural tract facilitating interhemispheric communication. This evolutionary convergence raises the possibility that the emergence of C14MC may have accompanied, or contributed to, the increasing complexity of mammalian cortical circuitry [59]. While this relationship remains speculative, it provides a compelling framework for considering C14MC not simply as a collection of independently acting miRNAs, but as part of an evolutionarily specialised regulatory architecture associated with cortical development.

Importantly, the C14MC members identified in the present study have not themselves been demonstrated to directly regulate corpus callosum development. Therefore, a direct mechanistic link between C14MC dysregulation and corpus callosum pathology cannot be inferred from these data. Nevertheless, their membership within an evolutionarily specialised cluster implicated in neuronal developmental programmes raises the possibility that some molecular pathways established during neurodevelopment may influence selective neuronal vulnerability later in life. This hypothesis is consistent with increasing evidence of structural and microstructural abnormalities of the corpus callosum across AD and FTD [60]. A possibility is therefore that alterations in C14MC-associated regulatory networks may represent a molecular intersection between developmental programmes, cortical connectivity and later-life neurodegeneration. Testing this hypothesis will require integration of developmental models, spatially resolved transcriptomic approaches and functional manipulation of individual C14MC members.

## 5. Conclusions

Although no consistent cluster-wide signature was detected, the limited sample size prevents broader C14MC alterations from being excluded. Nevertheless, the disease- and subtype-associated differences observed among individual C14MC miRNAs support further investigation of specific cluster members. Several individual miRNAs, including miR-758-5p, miR-323a-5p, miR-379-3p, miR-299-3p and miR-1247-5p, emerged as candidates warranting further investigation. Collectively, these findings establish a discovery framework for exploring BDsEV-associated C14MC miRNAs as molecular signatures of dementia and provide a basis for investigating whether this evolutionarily specialised miRNA cluster contributes not only to disease-associated biomarker profiles but also to the developmental and molecular mechanisms underlying selective neuronal vulnerability in neurodegeneration.

## Figures and Tables

**Table 1:** General demographic information of the frozen brain tissue samples within the study.

Sample Cohort	Sample Number (*n*)	Mean Age ± SE	Gender		APOE Variation					
			M	F	2/2	2/3	2/4	3/3	3/4	4/4
Control	5	89.4 ± 4.4	2	3	-	1	-	4	-	-
AD	5	76.4 ± 11.2	3	2	-	-	-	2	2	1
FTD-*GRN*	5	70.6 ± 2.7	3	2	-	-	-	5	-	-
FTD-*MAPT*	5	59.4 ± 4.2	1	4	-	-	-	5	-	-
FTD-*C9orf72*	5	71.0 ± 7.0	2	3	-	-	-	5	-	-
*Total*	*25*	*73.3 ± 4.9*	*11*	*14*	*-*	*1*	*-*	*21*	*2*	*1*

## Data Availability

The data supporting the findings of the current study are available from the corresponding author upon reasonable request. Additionally, code is available from the corresponding author upon reasonable request.

## Supplementary Materials

The following supporting information can be downloaded at: https://www.mdpi.com/article/10.3390/biom16101468/s1, Figure S1: Full western blot images of both negative and positive characterisation marker of BDsEVs; Figure S2: Native plots taken directly from ZetaView output files; Table S1: Concentra-tions and dilution factors of the antibodies used in western blotting characterisation of isolated brain-derived extracellular vesicles; Table S2: Forward primer sequences designed for the chro-mosome 14 microRNA cluster candidates for RT-qPCR experiments; Table S3: Endpoint PCR cy-cling conditions; Table S4: Composite C14MC expression scores across diagnostic groups; Table S5: Kendall’s coefficient of concordance (W) for C14MC candidate miRNA expression within each group; Table S6: One-Way ANOVA comparing relative expression of each C14MC candidate miRNA across diagnostic groups; Data S1: Donor Level Information; Data S2: RT-qPCR Results; Data S3: Count Matrix; Data S4: DESeq2 Results; File S1: C14MC Analysis R Script.
