# Supplementary material for: Chromosome 14 MicroRNA Cluster Members in Brain-Derived Extracellular Vesicles Can Be Genotype-Associated Biomarker Candidates for Alzheimer’s Disease and Frontotemporal Dementia
Source: Biomolecules. 2026 Oct 9;16(10):1468. doi: 10.3390/biom16101468 (PMC13702403; doi:10.3390/biom16101468)
Supplement: Supplementary file 1 [file biomolecules-16-01468-s001.zip › SupplementaryFigures_S1-S2.pdf]

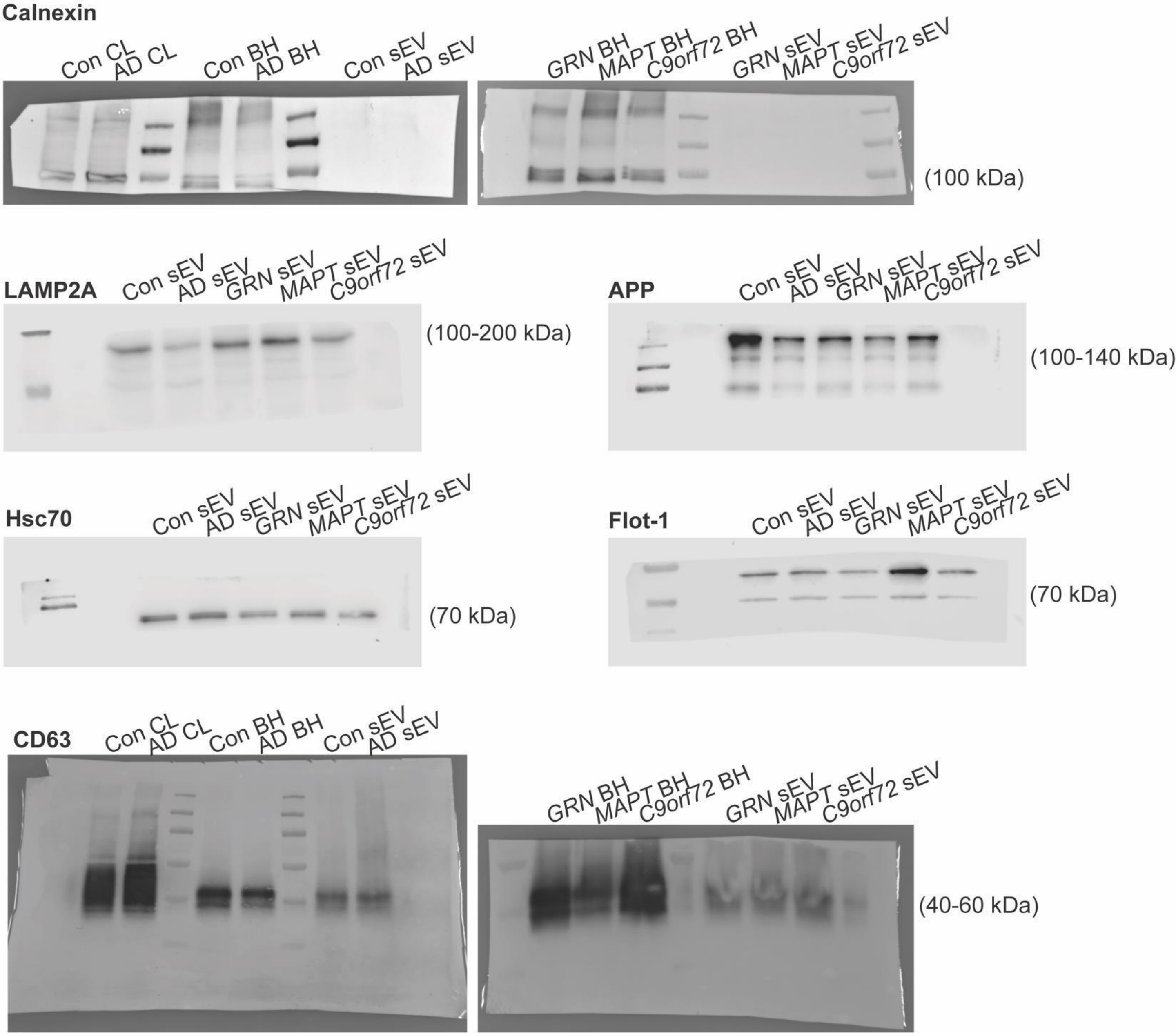

Figure S1. Full western blot images of both negative and positive characterisation markers of BDsEVs. The presence of the negative Calnexin is shown within the whole BH samples, yet absent within the isolated BDsEVs in all diagnostic group samples. The presence of LAMP2A, APP, Hsc70, Flot-1 and CD63 are displayed within all BDsEV samples, where only CD63 is shown ran against a positive BH sample.

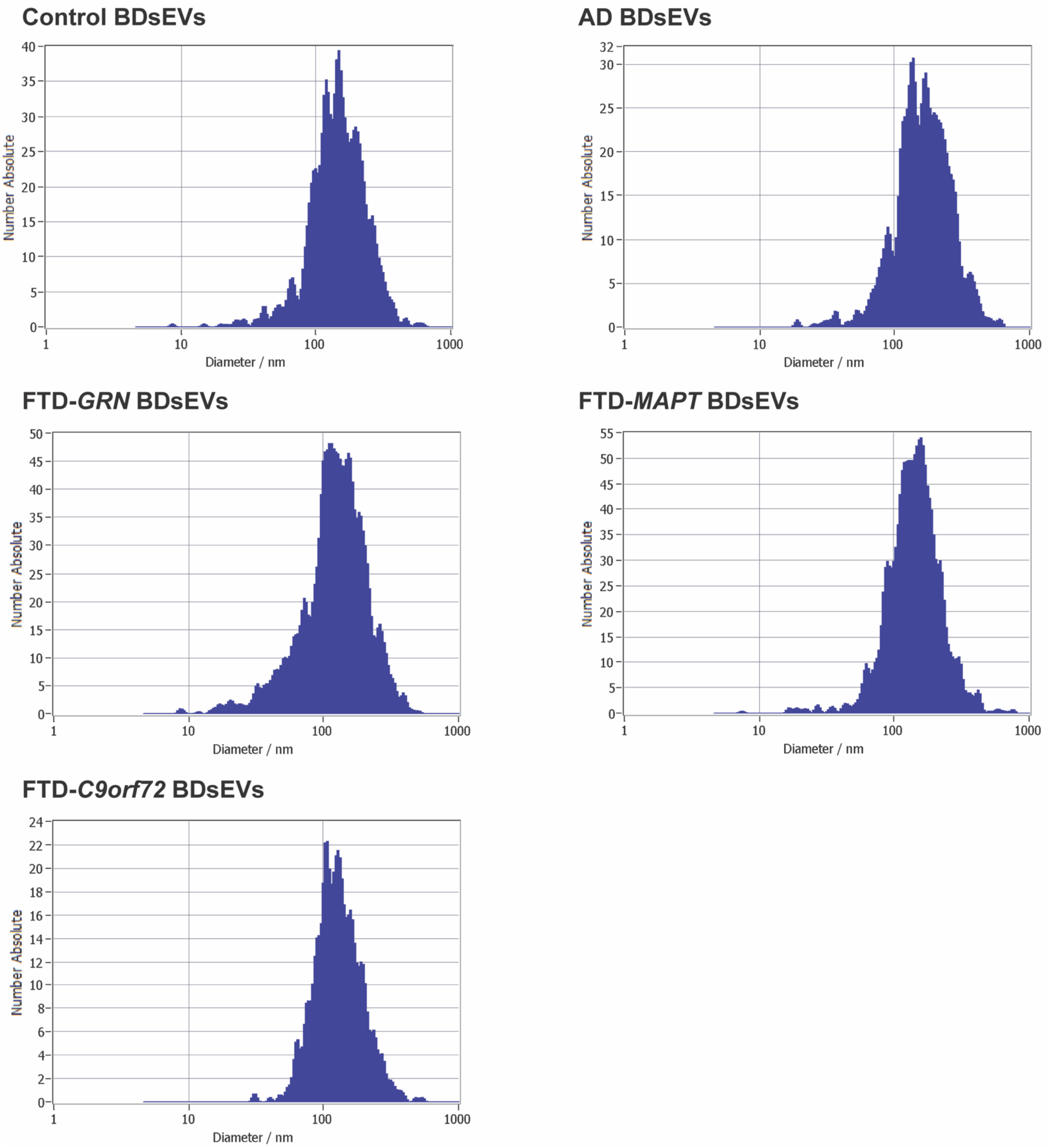

Figure S2. Native plots take directly from ZetaView output files. Plots (Number Absolute against Diameter in nm) are shown from one replicate of control, AD, FTD-*GRN*, FTD-*MAPT* and FTD-*CSorf72*.
