# Supplementary material for: Chromosome 14 MicroRNA Cluster Members in Brain-Derived Extracellular Vesicles Can Be Genotype-Associated Biomarker Candidates for Alzheimer’s Disease and Frontotemporal Dementia
Source: Biomolecules. 2026 Oct 9;16(10):1468. doi: 10.3390/biom16101468 (PMC13702403; doi:10.3390/biom16101468)
Supplement: Supplementary file 1 [file biomolecules-16-01468-s001.zip › SupplementaryTables_S1-S6.pdf]

Table S1. Concentrations and dilution factors of the antibodies used in western blotting characterisation of isolated brain-derived extracellular vesicles.

Characterisation Antibody	Size (kDa)	Company	Final Concentration (µg/ml)	Dilution Factor
Primary mouse anti-human GM130 Monoclonal Antibody (6662-1-Ig)	130	Proteintech	0.2	1:5000
Primary mouse anti-APP Monoclonal Antibody (60342-1-Ig)	100	Proteintech	1	1:1000
Primary mouse anti-human Calnexin Monoclonal Antibody (66903-1-Ig)	90	Proteintech	0.1	1:10000
Primary mouse anti-human CD63 Monoclonal Antibody (10628D)	40-60	Invitrogen	0.25	1:2000
Primary mouse anti-human Flotillin 1 Monoclonal Antibody (67968-1-Ig)	47	Proteintech	0.5	1:2000
Primary mouse anti-human CD81 Monoclonal Antibody (10630D)	25	Invitrogen	0.25	1:2000
Primary mouse anti-human CD9 Monoclonal Antibody (10626D)	25	Invitrogen	0.25	1:2000
Secondary goat anti-mouse IgG, HRP-linked Antibody (#7076)	-	Cell Signalling Technology	-	1:5000

Table S2. Forward primer sequences designed for the chromosome 14 microRNA cluster candidates for RT-qPCR experiments. Using miRBase (https://www.mirbase.org/), forward primers were designed and ordered from Eurofins Scientific, for use within the MasterMix. Alongside the sequence of the miRNA, GC content (%), melting point (°C) and molecular weight (g/mol) of each primer is provided.

MicroRNA	Sequence (5' - 3')	GC (%)	Tm (°C)	MW (g/mol)
hsa-miR-376a-2-5p	GGUAGAUUUUCCUUCUAUGGU	38	54	6433.2
hsa-miR-376c-5p	GGUGGAUAUUCCUUCUAUGUU	38	54	6433.2
hsa-miR-889-5p	AAUGGCUGUCCGUAGUAUGGUC	50	60.3	6781.4
hsa-miR-410-5p	AGGUUGUCUGUGAUGAGUUCG	48	62.1	6523.2
hsa-miR-323a-5p	AGGUGGUCCGUGGCGCGUUCGC	73	69.6	6799.4
hsa-miR-758-5p	GATGGTTGACCAGAGAGCACAC	55	57.9	6793.5
hsa-miR-134-5p	UGUGACUGGUUGACCAGAGGGG	59	64	6800.4
cel-miR-39	UCACCGGGUGUAAAUCAGCUUG	50	60.3	6750.4

Table S3. Endpoint PCR Cycling Conditions.

Event	Temperature (°C)	Time
Initial Denaturation	98	30 s
Annealing Cycle (22 Cycles)	98	10 s
	60	30 s
	72	15 s
Final Extension	72	10 min

Table S4. Composite C14MC expression scores across diagnostic groups.

Diagnostic Group	n	Mean	SD	Median	IQR
**Control**	3	-0.08473	0.28716	-0.18884	0.27264
**AD**	3	-0.24318	0.35966	-0.44757	0.31309
**FTD-*GRN***	3	-0.15100	0.83713	-0.07603	0.83461
**FTD-*MAPT***	3	-0.03105	0.15741	-0.06558	0.15454
**FTD-*C9orf72***	3	0.50995	0.36841	0.54790	0.36694
**One-Way ANOVA**	**df**	**Sum Sq**	**Mean Sq**	***F* Value**	***P* Value**
	4	1.05036	0.26259	1.22351	0.36060
	10	2.14622	0.21462		

Table S5. Kendall's coefficient of concordance (*W*) for C14MC candidate miRNA expression within each diagnostic group.

Diagnostic Group	W	χ^2	P Value
**Control**	0.06122	0.85714	0.65144
**AD**	0.06122	0.85714	0.65144
**FTD-*GRN***	0.73469	10.28571	0.00584
**FTD-*MAPT***	0.02041	0.28571	0.86688
**FTD-*C9orf72***	0.24490	3.42857	0.18009

Table S6. One-Way ANOVA comparing relative expression of each C14MC candidate miRNA across diagnostic groups.

miRNA	DFn	DFd	*F* Value	*P* Value	Significance
**miR-134-5p**	4	10	0.69473	0.61251	ns
**miR-323a-5p**	4	10	20.6675	7.99E-05	***
**miR-376a-2-5p**	4	10	0.66177	0.63251	ns
**miR-376c-5p**	4	10	0.35034	0.83805	ns
**miR-410-5p**	4	10	1.21735	0.36283	ns
**miR-758-5p**	4	10	3.48565	0.04972	*
**miR-889-5p**	4	10	1.37447	0.31025	ns
